# Porous Perovskite LaNiO_3_ Nanocubes as Cathode Catalysts for Li-O_2_ Batteries with Low Charge Potential

**DOI:** 10.1038/srep06005

**Published:** 2014-08-08

**Authors:** Jian Zhang, Yubao Zhao, Xiao Zhao, Zhaolin Liu, Wei Chen

**Affiliations:** 1 Department of Chemistry, National University of Singapore, 3 Science Drive 3, 117543, Singapore; 2 Institute of Materials Research and Engineering (IMRE), Agency of Science, Technology, and Research (A*STAR), 3 Research Link, Singapore 117602, Singapore; 3 Department of Physics, National University of Singapore, 2 Science Drive 3, 117542, Singapore; 4 National University of Singapore (Suzhou) Research Institute, Suzhou, China

## Abstract

Developing efficient catalyst for oxygen evolution reaction (OER) is essential for rechargeable Li-O_2_ battery. In our present work, porous LaNiO_3_ nanocubes were employed as electrocatalyst in Li-O_2_ battery cell. The as-prepared battery showed excellent charging performance with significantly reduced overpotential (3.40 V). The synergistic effect of porous structure, large specific surface area and high electrocatalytic activity of porous LaNiO_3_ nanocubes ensured the Li-O_2_ battery with enchanced capacity and good cycle stability. Furthermore, it was found that the lithium anode corrosion and cathode passivation were responsible for the capacity fading of Li-O_2_ battery. Our results indicated that porous LaNiO_3_ nanocubes represent a promising cathode catalyst for Li-O_2_ battery.

Rechargeable lithium-oxygen batteries with remarkably high theoretical energy storage capacity have attracted significant attention due to their potential applications in electric vehicles^1^,^2^,^3^,^4^,^5^,^6^,^7^. It is predicted that the energy density of the Li-O_2_ battery is around 10 times higher than that of the current Li-ion battery^1^,^4^. A typical rechargeable Li-O_2_ battery cell comprises porous cathode, lithium anode, separator and Li^+^ conducting electrolyte. However, this system suffers from many challenges for practical applications, such as electrolyte instability, poor cycle stability and high overpotential^8^,^9^,^10^,^11^. All these problems are related to the sluggish oxygen evolution reaction (OER). The overpotential for charge process (i.e., OER) is up to 1.0–1.50 V, which is much higher than that for discharge process (0.30 V)^12^. To date, the most efficient OER catalysts are noble metals^13^,^14^. For example, Ru nanocrystal shows a good catalytic performance with a discharge-charge overpotential as low as about 0.37 V^15^. However, the scarcity and high cost of noble metals limit their large-scale applications. Therefore, it is highly desirable to develop non-precious metal catalysts for OER^16^,^17^,^18^,^19^,^20^,^21^,^22^.

Perovskite oxides (ABO_3_), which are widely used as catalysts for fuel cells and zinc-air batteries, recently have also been evaluated for Li-O_2_ batteries^23^,^24^,^25^,^26^,^27^,^28^. Y. L. Zhao and his colleagues developed hierarchical mesoporous perovskite La_0.5_Sr_0.5_CoO_2.91_ nanowires and obtained high capacity of 11059 mAh g^−1^ 29. J. J. Xu et.al used perovskite-based porous La_0.75_Sr_0.25_MnO_3_ nanotube as the cathode for Li-O_2_ battery and cycled the battery over 124 cycles at a 1000 mAh g^−1^ capacity limitation^30^. S. H. Yang and co-workers have systematically investigated the electrocatalytic activity of perovskite oxide through molecular orbital principle; they predicted that LaNiO_3_ possessed unique intrinsic activity for both oxygen reduction reaction (ORR) and OER among the perovskite type oxides^31^,^32^. In addition, porous materials have been demonstrated to show extra advantage in Li-O_2_ battery applications^17^,^29^,^30^. The porous structure can provide ideal pathway for oxygen transfer and electrolyte diffusion, as well as more catalytic active sites to promote the ORR and OER.

In this work, porous pervoskite LaNiO_3_ nanocubes were synthesised and employed as the cathode catalyst for Li-O_2_ battery. The as-prepared catalyst showed improved performance in both discharge and charge process. In particular, in charge process, the catalyst could significantly reduce the overpotential up to ~260 mV and ~350 mV compared with the LaNiO_3_ particles and commercial Vulcan XC-72 carbon (VX-72) electrodes at the current density of 0.08 mA cm^−2^. The charge voltage could be even decreased to 3.40 V at lower current density of 0.016 mA cm^−2^. The Li-O_2_ battery assembled by the porous LaNiO_3_ nanocubes as cathode catalyst also showed enchanced capacity and good cycle stability.

## Results

### Synthesis and characterization of porous LaNiO_3_ nanocubes

**Figure 1:**
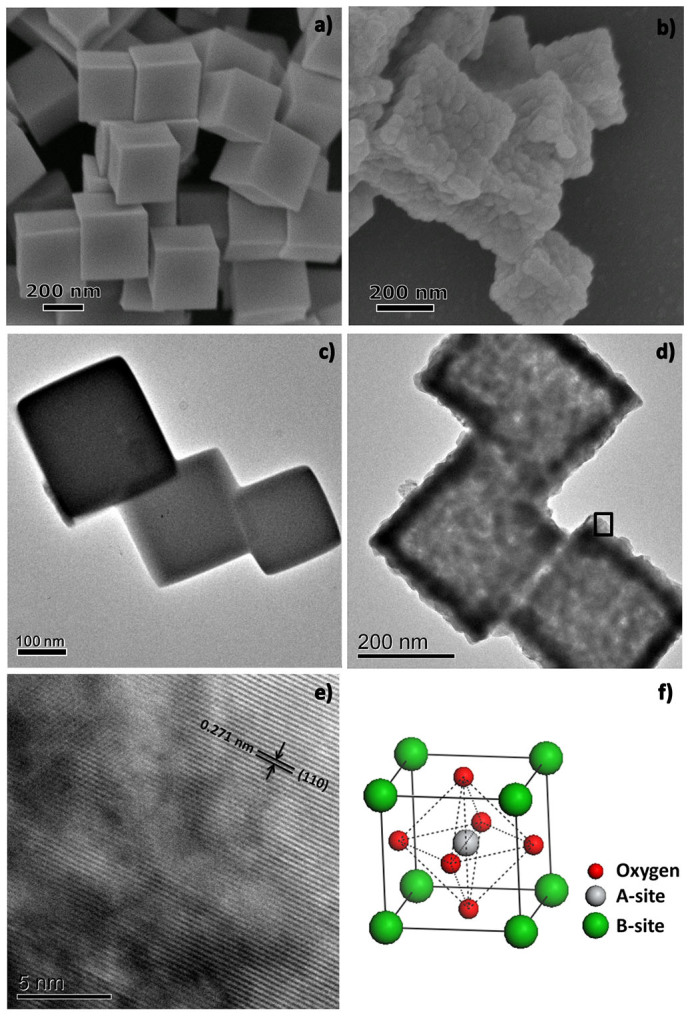
SEM (a, b) and TEM (c, d) images of the obtained nanocube-like precursors before (a, c) and after (b, d) annealing, respectively; (e) High-resolution TEM image of porous LaNiO_3_ nanocubes; (f) ABO_3_ perovskite oxides structure.

**Figure 2:**
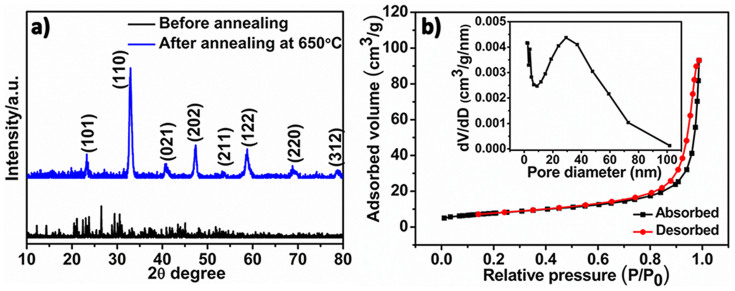
(a) XRD pattern of the nanocube-like precursors before(black) and after (blue) annealing; (b)Nitrogen adsorption-desorption isotherms and pore size distribution (inset) of porous LaNiO_3_ nanocubes catalyst.

The nanocube-like precursors were synthesized *via* a modified hydrothermal process^33^, with the pH value of 7.7 and the glycine to metal salt molar ratio of 3:1. Fig. 1a and 1c show the scanning electron microscopy (SEM) and transmission electron microscope (TEM) images of the as-prepared nanocube-like precursors. These precursors had smooth surfaces with the size about 250 nm. After annealing in O_2_ at 650°C for 2 h, the surface of the annealed products became rough and rich porosity was created (Fig. 1b and 1d). At the same time, the original cubic shape was not significantly changed. As displayed in the high-resolution TEM image in Fig. 1e, the distance of the adjacent fringes was 0.271 nm, corresponding to the lattice spacing of the (110) plane of perovskite-type LaNiO_3_. The X-ray diffraction (XRD) pattern (Fig. 2a) revealed that the annealed products were perovskite-type LaNiO_3_ (PDF#34-1028) without any La_2_O_3_ or NiO related phase. This indicates that the nanocube-like precursors had completely transformed into LaNiO_3_ after the 650°C annealing. The BET specific surface area of the annealed products was 35.8 m^2^ g^−1^ (Fig. 2b). It was nearly 10 times as high as that of the LaNiO_3_ particles prepared without glycine (Fig. S1). The average pore diameter of the porous LaNiO_3_ nanocubes was ~30 nm (Inset in Fig. 2b). However, without glycine, the nanocubic structure and rich porosity could not be obtained (Fig. S1, S2). Herein, glycine not only acted as a pore-forming agent but also a shape-control agent in the formation of porous nanocubic structure^34^,^35^.

### Electrochemical Measurements and Li-O_2_ Battery Test

**Figure 3:**
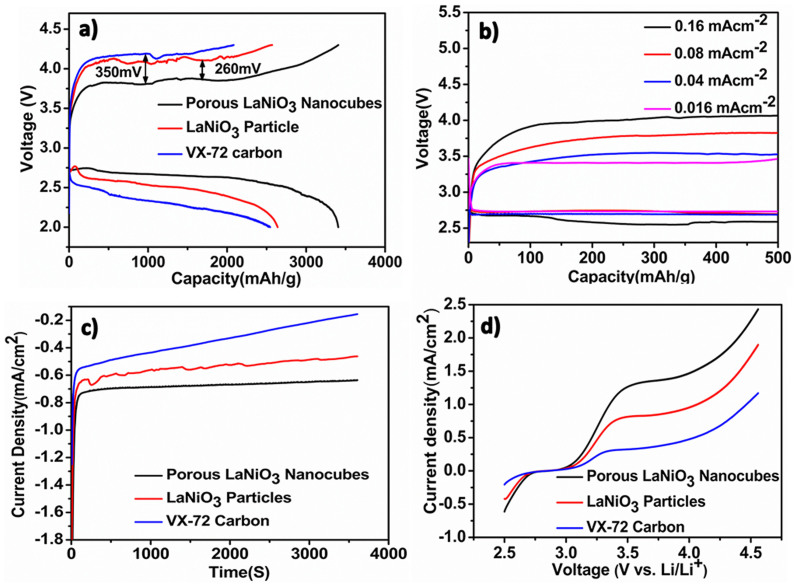
(a) First discharge-charge curves of Li-O_2_ batteries with porous LaNiO_3_ nanocubes, LaNiO_3_ particles and VX-72 carbon electrodes at 0.08 mA cm^−2^; (b) First discharge-charge curves of Li-O_2_ battery with porous LaNiO_3_ nanocubes electrode at 0.16, 0.08, 0.04 and 0.016 mA cm^−2^; (c) Chronoamperometry showing normalized current evolution with time for various catalysts at 2.25 V; (d) Linear sweep voltammetry of porous LaNiO_3_ nanocubes, LaNiO_3_ particles and Vulcan X72 carbon catalysts.

The electrochemical performance of the porous LaNiO_3_ nanocubes catalyst was measured by the galvanostatic charge-discharge measurements in a modified Swagelok Li-O_2_ battery cell using 1 M lithium trifluoromethanesulfonate/tetraethylene glycol dimethyl ether (LiCF_3_SO_3_/TEGDME) as the electrolyte. Reference cathodes made by either LaNiO_3_ particles or commercial VX-72 carbon were employed for comparison. Fig. 3a shows the first discharge-charge profiles of the Li-O_2_ cells with porous LaNiO_3_ nanocubes, LaNiO_3_ particles and VX-72 carbon electrodes at a current density of 0.08 mA cm^−2^. The discharge capacity of the battery cell with porous LaNiO_3_ nanocubes electrode was up to 3407 mAh g^−1^, which was higher than that of the LaNiO_3_ particles (2639 mAh g^−1^) and VX-72 carbon (2545 mAh g^−1^) electrodes based batteries. The enhanced discharge capacity of the porous LaNiO_3_ nanocubes electrode was attributed to their high catalytic activity to promote the ORR.

As shown in Fig. 3a, the porous LaNiO_3_ nanocubes catalyst possessed excellent OER performance with the charge voltage plateau at about 3.87 V, which was lower than that of the LaNiO_3_ particles and VX-72 carbon catalysts by ~260 mV and ~350 mV, respectively. In order to further investigate the OER performance of porous LaNiO_3_ nanocubes electrode, galvanostatic discharge-charge measurements at 0.16, 0.04 and 0.016 mA cm^−2^ were also performed (Fig. 3b). When the current density was 0.016 mA cm^−2^, the charge plateau was lower to 3.40 V. To our knowledge, this value was one of the lowest charge potentials among the reported metal oxide catalysts^36^,^37^,^38^. Even at a higher current density (0.16 mA cm^−2^), the charge plateau only increased to 4.00 V, which was also lower than that of other metal oxide catalysts at the same current density^17^,^30^. These results demonstrate that the porous LaNiO_3_ nanocubes catalyst possesses excellent OER performance under a wide range of current densities. Electrochemical measurement in non-aqueous electrolyte system was carried out to confirm the high OER performance of porous LaNiO_3_ nanocubes catslyst. As shown in chronoamperometry process and Linear sweep voltammetry (LSV) measurement (Fig. 3c and 3d), the porous LaNiO_3_ nanocubes catalyst showed higher response current density compared with LaNiO_3_ particle and VX-72 carbon catalysts, suggesting the promotion effects of porous LaNiO_3_ nanocubes catalyst on both ORR and OER^36^,^39^,^40^. The LSV measurement showed no obvious difference of the onset potential among porous LaNiO_3_ nanocubes, LaNiO_3_ particles and VX-72 carbon electrodes, which is consistent with previous reports^36^,^41^.

## Discussion

Our experimental results showed that the porous LaNiO_3_ nanocubes catalyst exhibited superior ORR and OER activity towards the formation and decomposition of discharge products, resulting in a low overpotential of the battery cell. The good catalytic activity of the porous LaNiO_3_ nanocubes electrode could be attributed to the intrinsic properties of perovskite-type LaNiO_3_^27^,^28^,^32^. Meanwhile, these catalysts also acted as a promoter to enhance the surface transport of LixO_2_ species by reducing their bonding strength with cathode materials in the discharge and charge process. This can largely faciliate the mass transport for both OR and OE^36^,^39^. In addition, the porous structure of the nanocubes benefited the fast and uniform diffusion of O_2_ and hence resulted in the uniform deposition and distribution of the discharge products (Fig. S3), thereby leading to the decomposition of the discharge products with low overpotential^42^.

**Figure 4:**
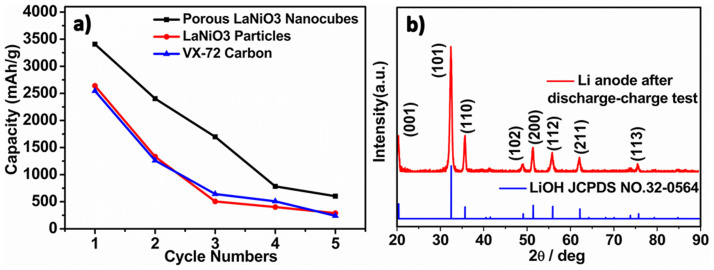
(a) Discharge capacity with cycle numbers under full capacity discharge-charge test at the current density of 0.08 mA cm^−2^; b) XRD pattern of the lithium anode after 3 discharge-charge cycles.

Although porous LaNiO_3_ nanocubes showed good ORR and OER performance, it was found that the Li-O_2_ battery cell suffered serious capacity fading during the full capacity discharge-charge tests. As can be seen in Fig. 4a, only 50% capacity was retained after 3 discharge-charge cycles. The serious capacity decay was usually ascribed to the electrode passivation, caused by partial blocking of active sites and pores by undecomposed Li_2_O_2_ and side products Li_2_CO_3_ during the discharge-charge cycles^37^,^43^,^44^,^45^. Interestingly, we also observed serious degradation at the Li anode. After 3 discharge-charge cycles the surface of the Li anode changed from metallic chip to white powders (Fig. S4), which was evidenced to be LiOH by the XRD measurement (Fig. 4b). The formation of LiOH coated on the anode can affect the cycle performance of the battery cell.

**Figure 5:**
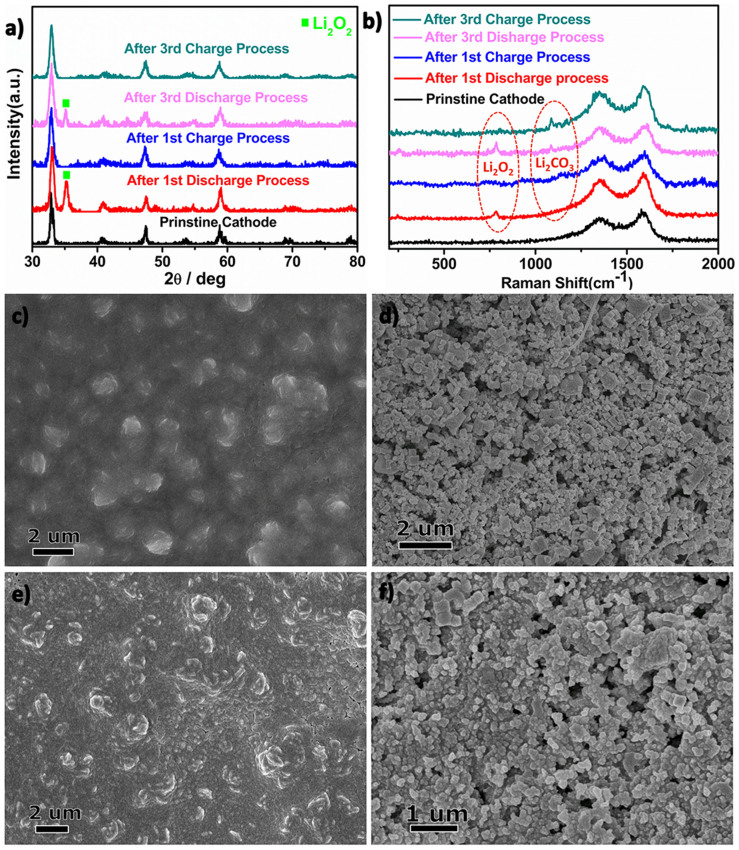
(a) XRD pattern of the electrodes at different states of discharge and charge; (b) Raman spectra of electrodes at different states of discharge and charge; SEM images of the cathode electrode after (c)1^st^ discharge, (d)1^st^ charge, (e)3^rd^ discharge and (f)3^rd^ charge, respectively.

To explore the origin of the capacity decay,Raman spectroscopy, XRD and SEM measurements were conducted to analyze the cathode at different discharge-charge states. After 1^st^ discharge, the discharge products coated on the surface of cathode homogeneously, as revealed by SEM image in Fig. 5c. These discharge products were evidenced to be Li_2_O_2_ based on the XRD pattern and Raman spectra (Fig. 5a and 5b)^47^,^48^,^49^. After charge, the Li_2_O_2_ related peaks disappeared from the XRD pattern and Raman spectra (Fig. 5a and 5b), indicating that all Li_2_O_2_ was decomposed without any obvious Li_2_O_2_ residual. The morphology of the cathode surface after the 1^st^ charge process (Fig. 5d) almost resembled that of the pristine electrode (Fig. S5), further confirming the complete decomposition of Li_2_O_2_ during the charge process. Similar trend was also observed by XRD and SEM measurement after the 3^rd^ discharge-charge cycle (Fig. 5a, 5e and 5f). However, a weak Li_2_CO_3_ peak was observed in the Raman spectra after 3^rd^ cycle (Fig. 5b). The Li_2_CO_3_ was proposed to originate from the unavoidable decomposition of electrolyte and unstable carbon components^50^,^51^. These Li_2_CO_3_ could block the small pores in the cathode, which can cause the cathode passivation and result in the capacity fading.

It was also found that the widely used polypropylene (PP) separators could not effectively prevent O_2_ diffusion to the Li anode, due to their large pore size (Fig. S6). Such oxygen crossover through the separators can promote the decomposition of electrolyte to form hydroxide and then react with lithium cations to form LiOH layer^46^. The trace amount of water diffused from the air and the moisture possibly existed in the electrolyte could also induce the formation of LiOH at the Li anode. The coating of the indecomposable LiOH on Li anode could greatly inhibit the discharge reaction^52^. Hence, the battery capacity fading was also caused by the incomplete recovery of the lithium anode during the charge process and the continuous consumption of Li by the formation of the indecomposable LiOH during the cycles. When “rebuilt” the battery cell by replacing the faded Li electrode with a fresh Li anode, an increased discharge capacity about 1500 mAh g^−1^ was obtained (Fig. S7). This result further confirmed that the capacity decay was partly originated from the corrosion of Li anode.

**Figure 6:**
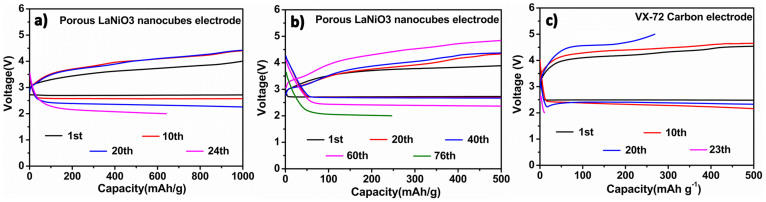
(a), (b) Cyclic performance of porous LaNiO_3_ nanocubes electrode at 0.08 mA cm^−2^ with limited capacity of 1000 mAh g^−1^ and 500 mAh g^−1^, respectively; (c) Cyclic performance of VX-72 carbon electrode at 0.08 mA cm^−2^ with limited capacity of 500 mAh g^−1^.

To minimize the side effects caused by anode corrosion and cathode passivation, we measured the Li-O_2_ battery cell with porous LaNiO_3_ nanocubes electrode following a recently widely adoped method^53^,^54^ by limiting the depth of discharge and “rebuilt” the cell with fresh Li metals during the test process. Improved cycle performance of Li-O_2_ battery was obtained. Fig. 6a shows the voltage profiles of porous LaNiO_3_ nanocubes electrode cycled at 0.08 mA cm^−2^ with the curtailing capacity of 1000 mAh g^−1^. The discharge-charge cycling was carried out for 23 cycles without abvious capacity fading (Fig. 6a). Reducing the curtailing capacity to 500 mAh g^−1^, the cycle number of the battery could increase to 75 cycles (Fig. 6b), which was much larger than that of VX-72 carbon electrode with 22 cycles under the same condition (Fig. 6c). These results indicate that the porous LaNiO_3_ nanocubes electrodes have good rechargeability and cyclability.

In summary, porous LaNiO_3_ nanocubes with large specific surface area were employed as cathode catalyst for Li-O_2_ battery. The battery assembled with porous LaNiO_3_ nanocubes electrode showed excellent charging performance with significantly reduced overpotentials (3.40 V). It also showed enhanced capacity of 3407 mAh g^−1^ and good cycle stability of 75 cycles without any obvious capacity decay at a 500 mAh g^−1^ capacity limitation. It was also found that the lithium anode corrosion and cathode passivation were responsible for the capacity fading of Li-O_2_ battery. This work suggests an alternative approach to develop high performance catalysts in Li-O_2_ battery by tuning the structure of perovskite oxides.

## Methods

### Synthesis of porous LaNiO_3_ nanocubes and LaNiO_3_ particles

Typically, 0.455 g La(NO_3_)_3_, 0.305 g Ni(NO_3_)_2_·6H_2_O and 0.394 g glycine were dissolved in 75 ml deionized (DI) water to form a transparent solution. The pH of the solution was then adjusted to about 7.7 by slowly adding NH_3_·H_2_O. After 10 min stirring, the solution was transferred into a 90 ml Teflon-lined stainless-steel autoclave and heated at 180 °C for 12 h. The obtained precursors were washed several times and dried at 80 °C. After that, the precursors were annealed at 650 °C for 2 h in O_2_ atmosphere to obtain porous LaNiO_3_ nanocubes. The reference LaNiO_3_ particles were synthesized with the same procedure without using glycine in the hydrothermal reaction system.

### Characterizations

The morphology and structure of the catalyst were characterized by SEM on JEOL JSM6700F and TEM on JEOL 3010, respectively. XRD patterns of the catalysts were recorded from a PANalytical Empyren DY 708 diffractometer with Cu radiation (Cu Kα = 0.15406 nm). BET surface area was measured by nitrogen sorption at 77 K on a surface area analyzer (QuadraSorb SI). Raman spectroscopy was carried out by the Renishaw Invia system with a 532 nm excitation line. The electrodes were washed with CH_3_CN in glove box before the ex-situ SEM, XRD and Raman spectroscopy analysis.

### Li-O_2_ cell assembly

The oxygen cathodes were prepared by coating the catalyst slurry on carbon paper homogenously. The catalyst slurry was prepared by mixing 40% porous LaNiO_3_ nanocubes or LaNiO_3_ particles catalysts with 50% VX-72 carbon and 10% polyvinylidene fluoride (PVDF), or 90% VC-72 carbon with 10% PVDF. The geometric area of the electrode was about 1.25 cm^2^ and the mass loading of the catalyst slurry on the electrode was about 1 mg cm^−2^. The capacity was calculated based on the total weight of electrode. All Li-O_2_ batteries were assembled using modified Swagelok cells in glove box under Argon atmosphere. The cell consisted of a lithium chip as the anode, polypropylene (PP) membrane as the separator and an as-prepared oxygen cathode. 1 M lithium trifluoromethanesulfonate/tretraethylene glycol dimethyl ether (LiCF_3_SO_3_/TEGDME) was used as the electrolyte. The galvanostatic discharge-charge test was conducted within a voltage window of 2.0–4.3 V (vs. Li/Li^+^).

### Rotating disk electrode (RDE) measurement in non-aqueous electrolyte

The catalyst ink was prepared by homogeneously dispersing 3 mg porous LaNiO_3_ nanocubes catalysts, 4.5 mg VX-72 carbon into 60 μl Nafion and 600 μl DI water solution. The thin-film electrode was then prepared by drop-casting the catalyst ink onto the flat GC electrode, yielding carbon loading of 0.3 mg_carbon_ cm^−2^_disk_. The RDE measurement system in non-aqueous electrolyte included a lithium-foil as the counter electrode, a reference electrode and a thin-film working electrode. The reference electrode was a silver wire immersing into 0.1 M tetrabutylammonium hexafluorophosphate (TBAPF_6_) and 0.01 M AgNO_3_ in TEGDME. Before using it was calibrated against Li metal in 1 M LiCF_3_SO_3_ TEGDME [0 V (vs. Li/Li^+^) ≈ −3.53 ± 0.01V (vs. Ag/Ag^+^)]. Prior to each experiment, the sealed test system was first purged with Ar for 15 mins, then the working electrode was cycled in Ar [3.4-2 V (vs. Li/Li^+^) at 100 rpm] until a stable cyclic voltammetric profile was obtained. Subsequently, the solution was purged with O_2_ for 30 mins for the chronoamperpmmetry studies, by holding the voltage at 2.25 V for 1 h to deposit Li_2_O_2_. Linear sweep voltammetry (LSV) measurement was conducted with potential scanning from 2.5 to 4.5 V (vs. Li/Li+) with the scan rate of 10 mV S^−1^.

## Author Contributions

J.Z. and W.C. designed the experiments. J.Z. performed the experiments. Y.B.Z. and X.Z. discussed and commented on the experiments and results. J.Z., Z.L.L. and W.C. discussed and wrote the paper.

## Supplementary Material

Supplementary Information supporting information
