# Supplementary material for: Porous Perovskite LaNiO3 Nanocubes as Cathode Catalysts for Li-O2 Batteries with Low Charge Potential
Source: Sci Rep. 2014 Aug 8;4:6005. doi: 10.1038/srep06005 (PMC4125986; doi:10.1038/srep06005)
Supplement: Supplementary Information — supporting information [file srep06005-s1.pdf]

Supporting information

Porous Perovskite $LaNiO_3$ Nanocubes as Cathode Catalysts for Li-O_2 Batteries with Low Charge Potential

Jian Zhang[1], Yubao Zhao[1], Xiao Zhao[1], Zhaolin Liu[2]* & Wei Chen[1,3,4]*

[1]Department of Chemistry, National University of Singapore, 3 Science Drive 3, 117543, Singapore

[2]Institute of Materials Research and Engineering (IMRE), Agency of Science, Technology, and Research (A*STAR), 3 Research Link, Singapore 117602, Singapore.

[3]Department of Physics, National University of Singapore, 2 Science Drive 3, 117542 Singapore

[4]National University of Singapore (Suzhou) Research Institute, Suzhou, China

Correspondence and requests for materials should be addressed to W.C (phycw@nus.edu.sg) Tel: (65) 6516-1879 FAX: (65) 6777-6126 and Z. L. L (zl-liu@imre.a-star.edu.sg)

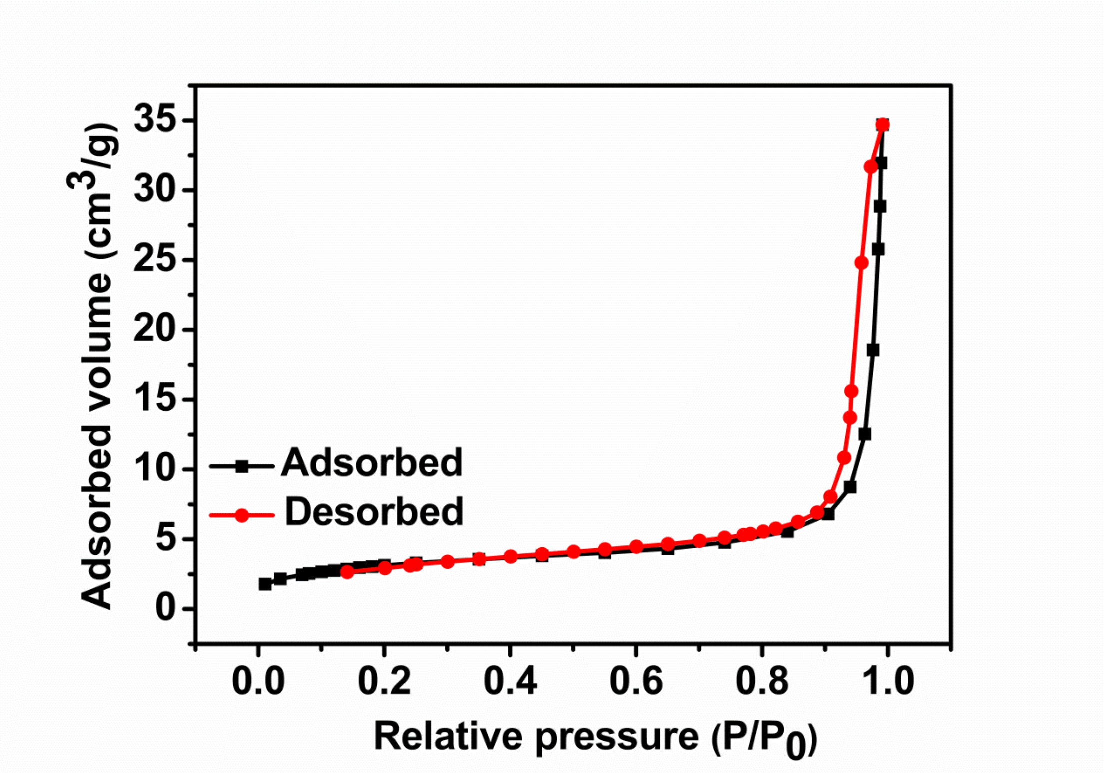

Figure S1. Nitrogen adsorption-desorption isotherms of $LaNiO_3$ particles. The BET surface area of the $LaNiO_3$ particles is about 4.7 m^2 g^{-1}. No pore structure was detected.

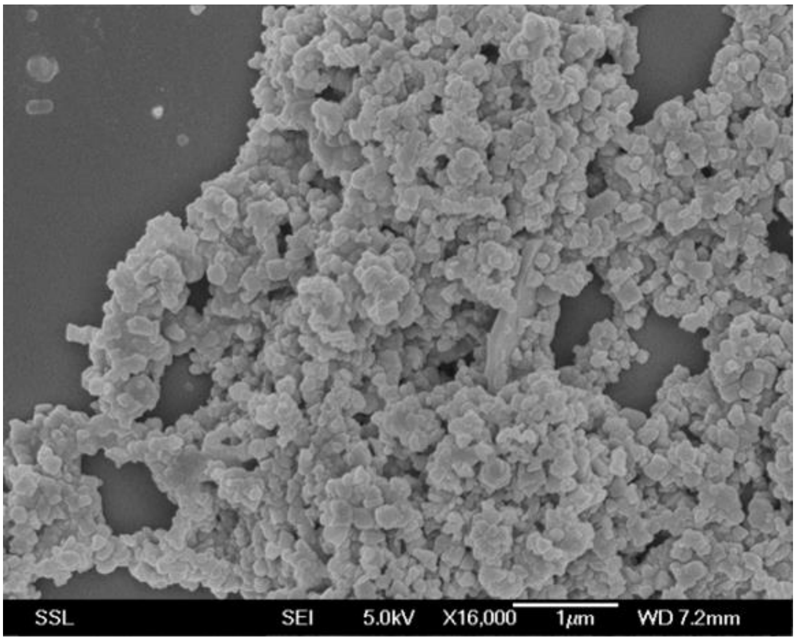

Figure S2. SEM image of $LaNiO_3$ particles

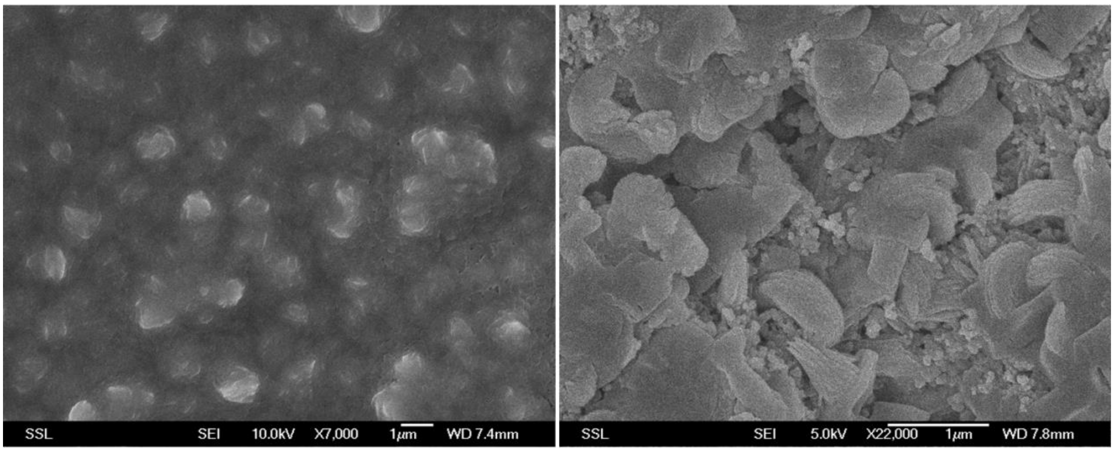

Figure S3. SEM image of the (a) porous $LaNiO_3$ nanocubes electrode and (b) VX-72 Carbon electrode after 1st discharge process at the current density of 0.08 mA cm^{-2}

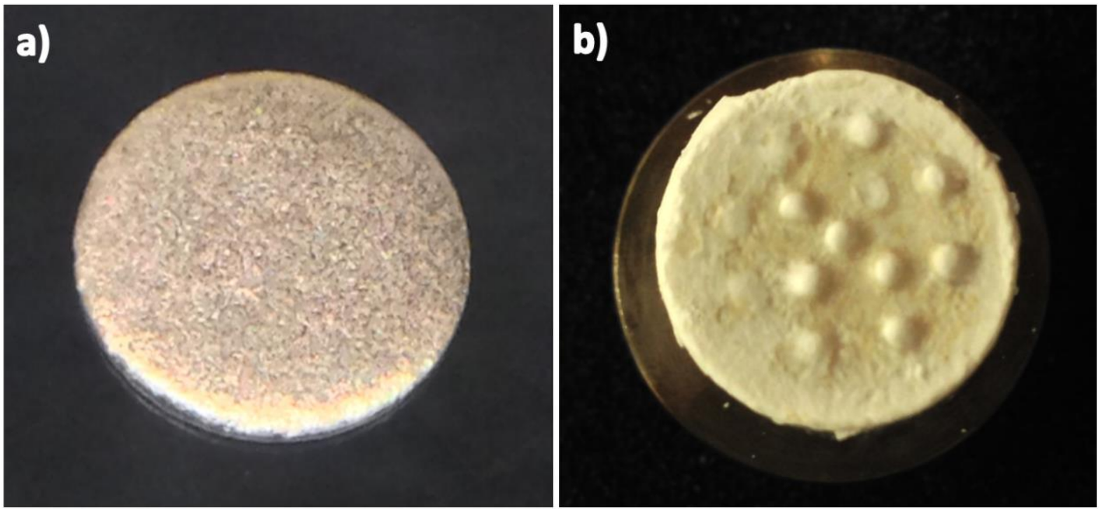

Figure S4. Optical images for (a) Lithium anode before test; (b) Lithium anode after 3 full capacity discharge-charge cycles

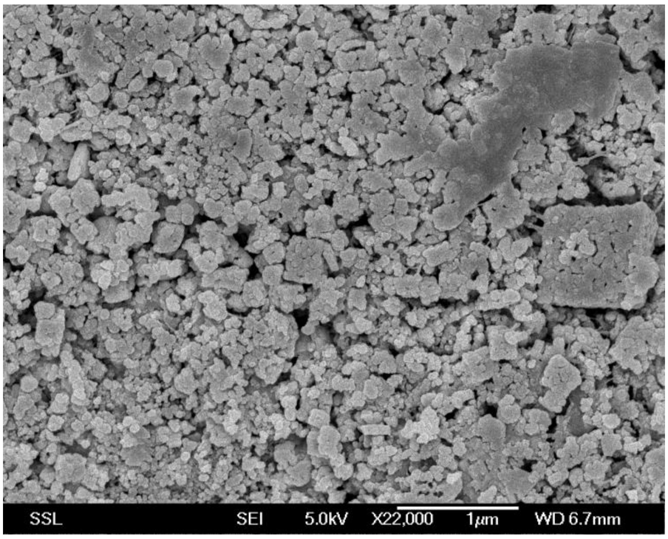

Figure S5. SEM image of the porous $LaNiO_3$ nanocubes electrode before test

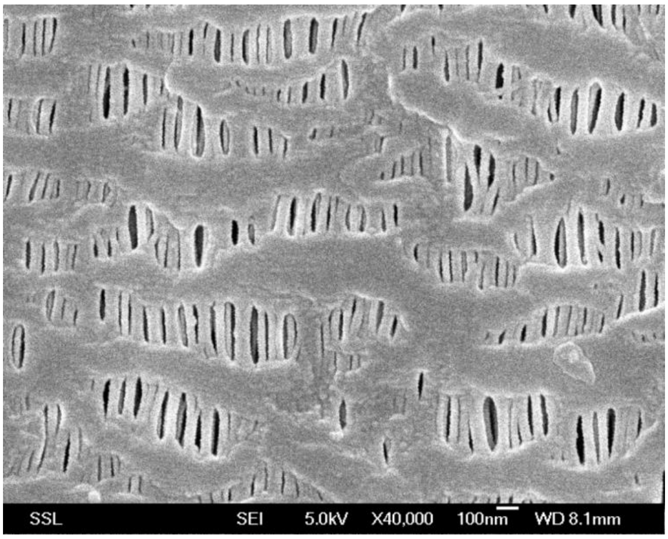

Figure S6. SEM image of monolayer polypropylene (PP) separator

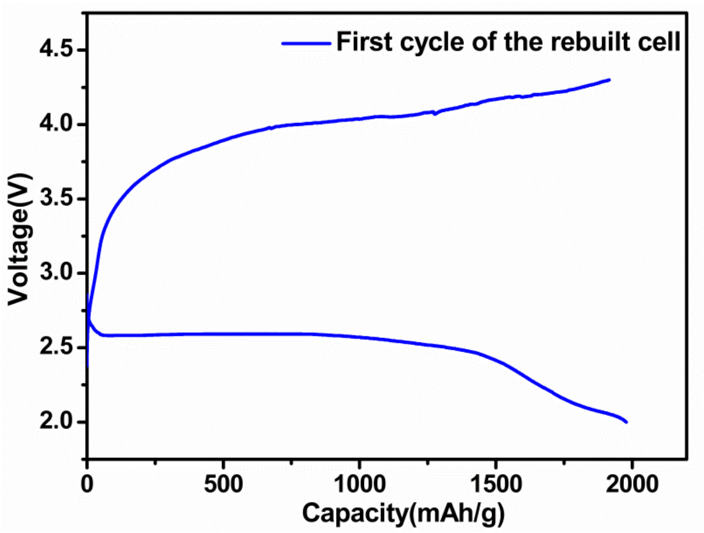

Figure S7. First discharge-charge curves of the "rebuilt" battery cell for porous $LaNiO_3$ nanocubes electrode at 0.08 mA cm^{-2}.
